# Reports on Hospitals of the United Kingdom

**Published:** 1914-05-09

**Authors:** Henry Burdett


### REPORTS ON

### Hospitals of the United Kingdom.

By SIR HENRY BURDETT, K.C.B., K.C.V.O.

SERIES III.

### WEST KENT GENERAL HOSPITAL, MAIDSTONE.

Maidstone may be regarded as the Metropolis of the Garden of England, but its voluntary hospitals are depressing indeed to visit. Its citizens, including the worthier men among them, seem to be asleep. In the England of to-day, at any rate, they stand out as people who have not yet grasped "the hospital idea," for to them sick people and sickness seem to make no awakening appeal. Our visit to the West Kent General Hospital on November 4, 1913, strengthened the impression we had had forced upon us that, speaking generally, few of the men of Kent have yet assimilated the parable of the Good Samaritan. Certainly it has not eaten its way into their hearts as it did into that of King Edward VII., who recognised, as his daily life proved, the privilege of personal service in the days of health in the cause of the sick.

### Ward Units and False Economy.

Here we have at Maidstone this general hospital established eighty years ago, conducted on almost the identical lines, so far as its committee is concerned, that were followed by the original founders. The West Kent General Hospital does not contain a single modern ward, nor has it a single ward unit properly equipped and sanitarily and hygienically complete. Some of the ward lavatories and soil sinks are nothing short of a disgrace to the managers who permit them to continue. There are no ward kitchens worthy of the name, and on one floor one bath is all that is provided for the use of the servants, of the nurses, and of the patients too! Further, and worse, if that be possible, there is no provision made for the proper keeping of such foods of various kinds as it is necessary to have, at times, near the wards for the patients. Another striking piece of evidence of the non-progressive character of the present managers, who, we have no doubt, are most excellent men, is that they appear to be impressed with the feeling that the main virtue of hospital committees is to refuse to spend money and continuously to hoard it. But what useful purpose this hoarding can fulfil, it is as impossible for us to say, as it is for them to demonstrate convincingly.

A voluntary hospital should be managed and brought up to the standard which will adequately meet the vital needs and claims of the people whose necessities it was established to supply. To do less than this is to fail of its purpose. In other words, hoarding money by swelling improperly the funds invested as a reserve, at a hospital of the Maidstone type, is so grave an evil, in our judgment, as to be equivalent to a distinct breach of trust on the part of a committee.

### The Reserve Fund.

When, as at the West Kent General Hospital, the invested funds, apart from special funds, exceed £30,000, whilst the average expenditure is some £5,000 a year, there is no justification for neglecting to spend money freely in making the equipment and management efficient. This Maidstone committee have more than £5,000 to the credit of the reserve fund in excess of the amount of £25,000, or five years' expenditure, which prudence requires that each voluntary hospital should keep invested against a rainy day. Yet the whole hospital is starved and neglected by a meanness that makes for inefficiency in all departments. Could this mistaken and wrong policy of the present committee go deeper than it has done? We think not, for the committee is well content, having ample funds in hand and accounts which practically balance each year, to occupy a board room, the meanness and disrepair of which are appalling, for they exhibit the low standard which appears to have eaten its way into the hearts of these managers so far as the upkeep and maintenance of their hospital are concerned.

### A Mistaken Policy.

The county of Kent appears to have invited subscriptions towards a memorial to King Edward VII. Upwards of £7,000 was raised and paid to the committee of the hospital, to be devoted to the erection of a new wing. The committee held that this sum was not large enough for the purpose, and so they invested it, and there the matter rests at present. We feel confident, and it is well to state the fact plainly in this connection, that had King Edward had a voice in the question of using this £7,000 for the benefit of this hospital as a memorial to himself, he would have commanded a sufficient portion of it to be expended at once in putting the present buildings into a proper state of completeness to enable the work of a modern hospital to be adequately carried out in the West Kent General Hospital, Maidstone. As it is, matters are allowed to remain, so far as lavatories and other portions of the ward units are concerned, in a state of insanitary and most discreditable neglect. Further, where new floors have been put down in some wards, instead of having them done thoroughly well in Canadian willow or teak, deal is used, from an idea of cheapness we suppose, to the discredit of the economic sense and reputation of those responsible for the order. The difference in cost between the best and the worst is relatively small, but we may perhaps take this use of condemned deal for hospital floors by this committee as evidence that they are convinced that an entirely new hospital for Maidstone must be built without delay. The most astonishing instance, however, of the miscalled economy practised by the present committee of the West Kent General Hospital is probably the letting off of the best portion of the relatively small garden, which is available for the patients' use to a tenant who pays, we believe, £5 a year for it. This portion is the orchard, and if it were kept in hand it could not fail to be a source of benefit to the patients, and might even yield most, if not all, of the £5 a year from the sale of the fruit.

### The Committee's Responsibility.

The present position of matters, so far as the hospital at Maidstone is concerned, is that, whereas the medical and surgical work being done is excellent, and that whereas the matron and the staff, though handicapped grievously by the non-expenditure of necessary money to equip the hospital properly in all its departments, are doing their duty faithfully and well, the reputation of the hospital and of the people of Maidstone is being lost. This calamity is owing to the absence of some man or men with eyes to see and knowledge to guide, with the fear of God sufficiently in them to realise the responsibility for every member of a committee of managers, who permits the hospital of one of England's greatest counties to languish and fall away from the high position it no doubt properly occupied in the days of their forebears and sires. There is not any single justification that we could discover for the present wantonly inefficient position, structurally, of this hospital. It is a marked discredit, serious and demonstrably provable, to every member of its present committee, and we feel it necessary to say with perfect frankness that they should either put their house in order or resign, leaving other and more public-spirited men to take the work in hand and make this hospital what it ought to be—*i.e.*, equal in efficiency and equipment to that of the best county hospital in the country.

The time has come when a new policy must be found and pursued by the responsible managers of the Maidstone Hospital. The affairs of the West Kent General Hospital have too long been allowed to remain in a rut, which may be taken as typical of the fact that the committee have somehow missed the truth. This is, that the character and amount of really good surgical and medical work now done at Maidstone represents a cost in material, in appliances and upkeep, which is infinitely greater than the cost twenty-five years ago. Against this extra outlay in a modern, up-to-date hospital has to be put the enormous value of the services rendered to the workers by these improved and more costly methods, which restore the patient at the earliest possible moment to his family in a condition to resume work and so to maintain them. Thus the earning power of the working classes in London each year has been increased to the extent of more than one million extra days' pay through the increased efficiency of the modern hospital.

### The Advertisement of Efficiency.

The only possible justification we can find for the Maidstone committee's attitude of indifference to efficiency and to many things the West Kent General Hospital needs, and ought to have forthwith, is, that they have never yet appreciated the power of efficiency in exciting interest in a hospital's work, in attracting to it new friends, and so materially increasing its prosperity in every way. We could wish that every inhabitant of Maidstone, and especially the members of the hospital committee, could have been with us during our inspection, and could have understood, as we understood, the real meaning of the excellent work done by the medical staff, and the loss to the sick and to the well-being of the people from the starvation policy so mistakenly and continuously pursued by the present managers.

The mere joy of rendering a little personal service in days of health in the cause of the sick becomes to every man and woman who has once experienced it a most precious possession, and secures that each will gladly make it his or her business to devote a definite portion of time each year to personal service in the cause of the sick. If we could only meet the people of Maidstone face to face and discuss the whole position with them as we see it and know it to be, we are confident that before six months were out the West Kent General Hospital would be made worthy of the highest traditions of our voluntary hospital system. The existing wards, as we have said, may for the most part, stand; but they must have isolation towers connected with them, and also properly planned and up-to-date ward kitchens, lavatories, and adequate bathrooms. The necessary reconstructions could be readily carried out, providing the hospital secured competent technical advice backed by practical and up-to-date knowledge of hospital construction and the essentials of a modern, up-to-date hospital.

The West Kent General Hospital does not seem to have been fortunate in securing such skilled assistance in the past, bearing in mind the manifest defects existing within it, as we have described them in this report.

### Finance.

We note with satisfaction that the committee have altered the date of the annual meeting to enable the accounts to be made up to December 31 in each year. We hope this step will be followed by the introduction of the Uniform System of Accounts, so that the statement of receipts and expenditure may be rendered in a form which will enable the management of this institution, from the economical and revenue standpoints, to be compared on its merits with other similar institutions. At present the income is returned with so much detail and with so little attempt at classification as to make the accounts confusing. There are two items in three years' accounts which call for criticism; and the present regular sale of stock could cease with sound financial management.

Undesirable Fees.

One entry records a portion of the rental of the garden already referred to as £5 a year, and the other apparently records for the first time receipts, from the nurses' and probationers' fees, amounting to £80. These fees ought not to have been levied seeing that the size of the hospital, which contains less than 100 beds, is not sufficient to enable the nurses to be properly trained or certificated at the end of three years' work at the institution, so as to enable them to rank as trained and certificated nurses. This is another reason why what we have ventured to describe as a system of discreditable economy should be promptly abandoned, a point to which we specially direct the attention of the managing committee and of the governors. The managers ought to take up this matter without a moment's avoidable delay and reconsider the present financial policy with the object of putting the hospital on a better, sounder, and more modern basis.

It is, indeed, unthinkable that the county of Kent should longer remain without a modern, wisely administered, properly constructed, ample hospital at Maidstone, or that it should longer suffer from a discredit which, in the hospital sense, attaches to the town of Maidstone. The funds available amply justify a bold and forward policy, so we beg the inhabitants to rouse themselves and to bring pressure to bear, if necessary, upon the present committee, that the good name and best traditions of the men of Kent may be steadily upheld and rescued from the discredit with which both are threatened by the state of affairs we have criticised in this report.

The Men of Kent and a Forward Policy.

A new and better policy must be adopted by the managers and governors of the West Kent General Hospital, in justice to the responsibilities, the wealth and collective prosperity of the men of Kent. Once the committee adopt a forward policy and provide Maidstone with an up-to-date and enlarged county hospital through a wisely conceived plan of reconstruction under competent expert advice, so as to prevent a repetition of the mistakes in construction which have characterised the additions to this hospital in the past, they may rely upon an increased income of sufficient amount to enable the West Kent General Hospital to be administered and maintained in the highest state of efficiency. This step, once taken, it should soon compare favourably with other county hospitals, instead of being, as at present, structurally and in matters of administration and business policy (though not in its medical and domestic management), so out of touch and behind the times in the requirements of modern science and hospital administration. Indeed, its present condition has fallen to so low a level as to make it rank at the very bottom of the list of provincial hospitals of its type.

Wake up, men of Kent! Regard this hospital question as a sacred responsibility in which every man and woman should take an active part, and everything requisite to give you a county hospital of the first rank can be readily and speedily accomplished.

